# Muscle and cerebral oxygenation during exercise in fibromyalgia: a near-infrared spectroscopy study

**DOI:** 10.1007/s00421-025-06013-8

**Published:** 2025-10-09

**Authors:** Taneli Lehto, Teemu Zetterman, Dominique Gagnon, Ritva Markkula, Jari Arokoski, Eija Kalso, Juha E. Peltonen

**Affiliations:** 1 Sports and Exercise Medicine, Faculty of Medicine, University of Helsinki, Mäkelänkatu 47, Urhea-hall, 00550 Helsinki, Finland; 2 Department of Anaesthesiology, Intensive Care and Pain Medicine, Helsinki University Hospital and University of Helsinki, Helsinki, Finland; 3 Wellbeing Services County of Vantaa and Kerava, Vantaa and Kerava, Finland; 4 Department of General Practice and Primary Health Care, Faculty of Medicine, University of Helsinki, Helsinki, Finland; 5 Helsinki Sports and Exercise Medicine Clinic (HULA), Foundation for Sports and Exercise Medicine, Helsinki, Finland; 6 Department of Internal Medicine and Rehabilitation, Division of Rehabilitation, Helsinki University Hospital and University of Helsinki, Helsinki, Finland; 7 SleepWell Research Programme, Faculty of Medicine, University of Helsinki, Helsinki, Finland

**Keywords:** Near-infrared spectroscopy, Fibromyalgia, Deoxygenation, Muscle oxygen saturation, Cardiopulmonary exercise test

## Abstract

**Purpose:**

We studied muscle and brain oxygenation during submaximal and peak exercise in patients with fibromyalgia (FM), a condition characterized by pain and fatigue, compared with age-matched healthy controls (CON).

**Methods:**

The participants (FM, n = 16; CON, n = 17) undertook a step incremental cycle spiroergometry until exhaustion. Changes in oxyhemoglobin (O_2_Hb), deoxyhemoglobin (HHb), total hemoglobin (tHB), and tissue saturation index (TSI%) using near-infrared spectroscopy were assessed in the vastus lateralis (leg) and biceps brachii (arm) muscles as well as the brain prefrontal cortex. Vastus lateralis blood flow (Q̇_VL_) was calculated from whole-body oxygen consumption (V̇O_2_) and HHb values. We analyzed between-group differences at absolute workloads and relative to V̇O_2peak_.

**Results:**

No significant between-group differences emerged in leg or arm oxygenation. Q̇_VL_ was lower in FM at 50% (0.066 [0.022] vs. 0.080 [0.028] a.u.), 75% (0.082 [0.020] vs. 0.106 [0.039] a.u.), and 100% (0.091 [0.036] vs. 0.111 [0.041] a.u.) of V̇O_2peak_, but similar at submaximal absolute workloads. Cerebral HHb values were higher in FM at 75 (1.09 [2.23] vs. −0.18 [1.38] μM) and 100 W (2.20 [2.96] vs. 0.06 [1.92] μM) but not at relative workloads. Cerebral TSI%, O_2_Hb and tHb were not significantly different between groups.

**Conclusion:**

Leg oxygenation or peak exercise cerebral deoxygenation in FM do not differ from controls. Q̇_VL_ was lower at peak exercise in FM. These results present evidence that exercise intolerance in FM may rely on other mechanisms than muscle oxygenation.

*Trial registration* ClinicalTrials.gov, NCT03300635. Registered 3 October 2017—retrospectively registered. https://clinicaltrials.gov/ct2/show/NCT03300635.

**Supplementary Information:**

The online version contains supplementary material available at 10.1007/s00421-025-06013-8.

## Introduction

Fibromyalgia (FM) is a condition characterized by persistent pain and fatigue. It is regarded as a prototype of nociplastic pain highlighting the importance of the central nervous system in its pathophysiology (Clauw 2024). However, in addition to top-down mechanisms where the central nervous system is the primary driver of pain, peripherally driven bottom-up mechanisms are also recognized (Sarzi-Puttini et al. 2020; Clauw 2024). Some authors have hypothesized that circulatory impairments (Kasikcioglu et al. 2006) and an imbalance between muscle oxygen supply and demand (Rubio-Zarapuz et al. 2025) may explain pain and fatigue in FM, but the evidence remains sparse. Impairments in mitochondrial function (Cordero et al. 2010; van Tilburg et al. 2020) and structure (Israel et al. 2024) as well as muscle (Gerdle et al. 2013) and brain (Jung et al. 2021) energy metabolism have also been reported using diverse methods but these findings have not been consistently replicated. We have previously demonstrated that patients with FM have lower peak oxygen uptake (V̇O_2peak_), cardiac output (Q̇) and arteriovenous oxygen difference (C(a-v)O_2_) suggesting that both oxygen delivery and extraction in the working muscles are decreased at peak exercise, whereas neither C(a-v)O_2_ nor Q̇ were impaired at submaximal workloads (Lehto et al. 2023). Understanding the mechanisms behind reduced exercise capacity is crucial for developing meaningful and effective rehabilitation interventions and novel therapeutic strategies.

Muscle energy transfer during exercise lasting longer than a few minutes is predominantly oxygen dependent (i.e. aerobic) (Hargreaves and Spriet 2020), where oxygen uptake increases to meet rising energy demand. Circulating oxygen is dissociated from hemoglobin in the capillaries, and transits into the muscle and mitochondria via diffusion (Wagner 2008). Exercise training increases muscle capillary density and vasodilation enabling more efficient oxygen extraction and widening of C(a-v)O_2_ (Poole et al. 2021). Muscle oxygenation can be studied with near-infrared spectroscopy (NIRS), but so far, limited scientific reports have utilized NIRS to assess muscle oxygenation in FM (Dinler et al. 2007, 2009; Shang et al. 2012; Srikuea et al. 2013; Schamne et al. 2024). Additionally, the results of these studies are not consistent, but early reports showed slower muscle oxygen recovery after an insult (ischemia or fatigue) (Dinler et al. 2007, 2009; Shang et al. 2012). A recent study investigating the effects of caffeine in FM patients found no differences in muscle oxygen saturation during constant cycling between patients and controls (Schamne et al. 2024). Interestingly, the main finding of the study was the central origin of muscle fatigue in FM (Schamne et al. 2024). Further research is needed to clarify the mechanisms behind muscle oxygenation and fatigue in FM and whether other mechanisms may also be at play.

Cerebral oxygenation might be one factor limiting exercise performance (Oussaidene et al. 2013). The prefrontal cortex (PFC) region of the brain is involved in the decision making of when to terminate exercise (Robertson and Marino 2016). PFC oxygenation during exercise is influenced by aerobic fitness and training (Oussaidene et al. 2015; Caen et al. 2019), although controversy exists (Buzza et al. 2016). Moreover, cerebral saturation during exercise is reduced in patients with heart failure and it correlates with V̇O_2peak_ and oxygen uptake efficiency (Chen et al. 2018). Low V̇O_2peak_ is widely documented in FM (Zambolin et al. 2022) and reduced volume of the PFC in FM has also been observed (Murillo-Garcia et al. 2022). Fatigue is a key symptom in FM (Zetterman et al. 2024), but it is not known to what extent subjective fatigue in FM is affected by PFC function. PFC oxygenation during exercise remains unstudied in FM patients.

In this study, we further investigated exercise responses in FM aiming to explore whether patients with FM have deviating muscle and cerebral oxygenation compared with healthy controls. Moreover, we wanted to assess peripheral blood flow in the exercising muscle. Previous studies indicate that vasoconstrictor signals efficiently oppose the vasodilatory metabolites in the arms, suggesting that during whole body exercise in the upright position blood flow is differentially regulated in the upper and lower extremities (Calbet et al. 2007). Moreover, brachial artery blood flow attenuates during exercise in conditions of added inspiratory resistance, likely due to increased sympathetic vascular tone (Katayama et al. 2019). Exercise induced changes in the autonomically regulated neurocardiovascular system have been sparsely studied in FM, but existing research on heart rate and heart rate variability suggests reduced sympathetic responses during maximal exercise (da Cunha Ribeiro et al. 2011; Schamne et al. 2021). Although distribution of cardiac output during exercise has not been explored in FM, the proposed attenuated sympathetic response may alter blood flow redistribution from inactive to active muscles, potentially affecting muscle oxygenation. Muscle oxygenation was thus assessed both in the exercising vastus lateralis (VL) as well as the relatively inactive biceps brachii (BB) muscle. Based on our previous findings on FM patients’ Q̇ and C(a-v)O_2,_ which were lower at peak but not at submaximal exercise, we hypothesized that working muscle deoxygenation would not differ between patients and controls at given submaximal work rates but would be lower in the FM group at peak exercise. In addition, we hypothesized that cerebral oxygenation would be compromised in FM due to lower cardiorespiratory fitness and peak Q̇.

The work presented here is part of a larger study; Metabolism, Muscle Function, and Psychological Factors in Fibromyalgia, where the participants also underwent electromyography and cognitive stress studies, metabolomic panel, and an oral glucose tolerance test in addition to a cardiopulmonary exercise test (Zetterman et al. 2021a, b, 2023, 2024; Lehto et al. 2023).

## Materials and methods

### Study participants

**Fig. 1:**
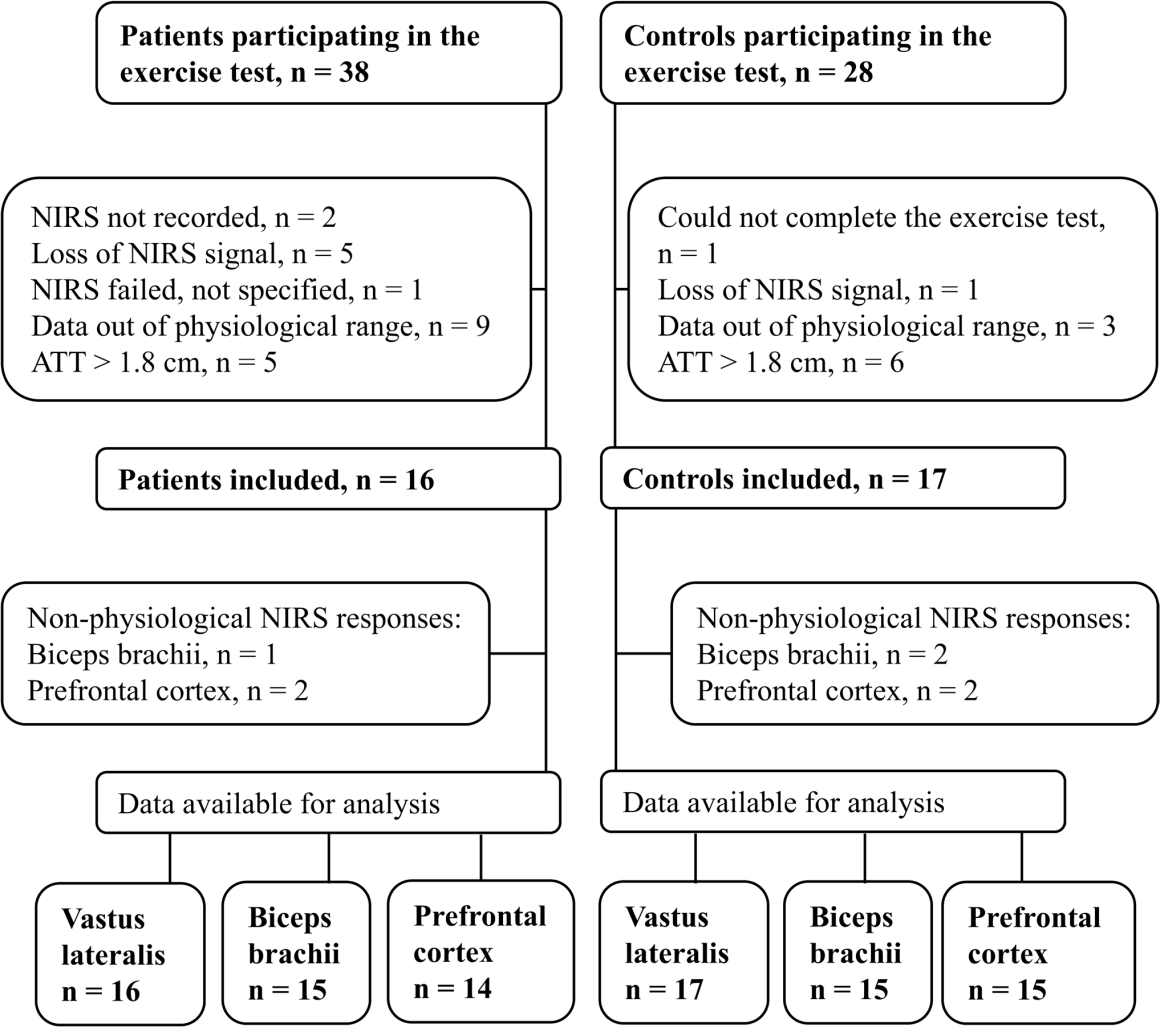
Flowchart of the participants. *ATT* adipose tissue thickness

Thirty-eight women with FM and twenty-eight age-matched healthy women (CON) undertook a cardiopulmonary exercise test until volitional fatigue. We recruited patients from Helsinki University Hospital outpatient clinics and primary healthcare centers in the City of Vantaa, Finland. We used the American College of Rheumatology 1990 Criteria for the Classification of Fibromyalgia for patient inclusion (Wolfe et al. 1990). The controls were recruited from the staff of the above-mentioned healthcare units and from a regional chapter of a home economics organization (The Martha Association). The initial recruitment process and exclusion criteria are described in detail in our previous publications (Zetterman et al. 2021b; Lehto et al. 2023). Briefly, cardiovascular, neurological or other diseases preventing participation in the exercise test were reasons for exclusion. FM severity was assessed with the Finn-FIQ (Finnish version of the Fibromyalgia Impact Questionnaire), the results of which were retrieved from a previous phase of the study (Zetterman et al. 2021b). After exclusions, 16 patients with FM and 17 controls from our previous study were analyzed. Participant exclusions are shown in Fig. 1. Exercise test results and participants background data are presented in Table 1.

**Table 1:** Participant data on demography and cardiopulmonary exercise test

	Fibromyalgia (*n* = 16)	Controls (*n* = 17)	*P*
Demographic data			
Age (years)	49 [15]	51 [11]	0.885^a^
BMI (kg·m^−2^)	25.8 ± 5.2	24.3 ± 4.1	0.358
Body fat (%)	33.8 ± 9.8	28.7 ± 8.5	0.123
Adipose tissue thickness, thigh (mm)	11 ± 4	12 ± 3	0.330
Adipose tissue thickness, arm (mm)	5 ± 2	4 ± 2	0.146
Smoking	4 (25)	1 (6)	0.175^b^
FIQ	36 ± 17	n/a	n/a
Cardiopulmonary exercise test			
Peak work rate (W)	109 ± 18	154 ± 35	< 0.001*
V̇O_2peak_ (mL∙min^−1^∙kg^−1^)	23.2 ± 6.0	31.3 ± 9.4	0.006*
RER_peak_	1.17 [0.16]	1.13 [0.08]	0.213
Q̇_peak_ (L∙min^−1^)	14.3 ± 1.1	15.3 ± 2.7	0.179
C(a-v)O_2peak_ (mL O_2_ 100 mL blood^−1^)	10.7 [3.1]	13.7 [6.8]	0.029^a*^
PETCO_2peak_ (mmHg)	35 ± 5	33 ± 3	0.089
MAP_peak_ (mmHg)	128 ± 18	132 ± 16	0.236
SVR_peak_ (mmHg∙min∙L^−1^)	7.9 ± 1.0	7.2 ± 1.4	0.162

Parametric data expressed as mean ± SD, nonparametric data as median [interquartile range], and categorical data as count (%). *P* values refer to unpaired t-test, except for a, refers to Mann–Whitney U test, and b, refers to Pearson *Χ*^*2*^ or Fisher’s Exact Test. *, *P* < 0.05. C(a-v)O_2_ and SVR data missing for one participant in the FM and three in the control group. MAP data missing for one participant in both groups. *BMI* body mass index, *FIQ* fibromyalgia impact questionnaire, *V̇O*_*2*_ oxygen uptake, *RER* respiratory exchange ratio, *Q̇* cardiac output, *C(a-v)O*_*2*_ arteriovenous oxygen difference, *PETCO*_*2*_ partial pressure of end-tidal carbon dioxide, *MAP* mean arterial pressure, *SVR* systemic vascular resistance

### Cardiopulmonary exercise test

We used a step incremental cycle ergometry (Monark Ergomedic 839E; Monark Exercise AB, Vansbro, Sweden) protocol with an initial 5 min of seated rest followed by 5 min of unloaded cycling after which the workload increased with by 25 W increment every 3 min until volitional fatigue. Breath-by-breath ventilation (V̇E), alveolar gas exchange including oxygen uptake (V̇O_2_), carbon dioxide production (V̇CO_2_), and partial pressure of end-tidal carbon dioxide (PETCO_2_), were measured (Triple V; Jaeger Mijnhardt, Bunnik, the Netherlands; AMIS 2000; Innovision A/S, Odense, Denmark), and respiratory exchange ratio (RER) was calculated. We used an impedance cardiography (ICG) device (PhysioFlow; Manatec Biomedical, Paris, France) to measure Q̇ and together with diastolic and systolic blood pressure from the brachial artery (Tango +, SunTech Medical, Morrisville, NC, USA) to calculate mean arterial pressure (MAP) and systemic vascular resistance (SVR). Systemic C(a-v)O_2_ was solved from the Fick principle, C(a-v)O_2_ = V̇O_2_/Q̇. Detailed methods and results of the cardiopulmonary exercise test have previously been published (Lehto et al. 2023) and the data presented here have been collected during the same exercise tests.

### Muscle and cerebral tissue (de)oxygenation

Near-infrared spectroscopy (NIRS) is a noninvasive method for measuring changes in tissue’s oxygenated (O_2_Hb) and deoxygenated hemoglobin (and myoglobin) (HHb) concentrations. In addition, total hemoglobin (tHb, O_2_Hb + HHb) and tissue saturation index (TSI%, O_2_Hb/tHb * 100%) can be calculated. The theoretical basis as well as inherent limitations of the method have been discussed elsewhere (Barstow 2019). Briefly, NIRS is based on the absorption and scattering of light in the investigated tissue. In skeletal muscle, the main absorbing chromophores are hemoglobin and myoglobin alongside cytochrome oxidase. The absorption properties of hemoglobin vary depending on whether it has oxygen bound to it or not, and O_2_Hb and HHb can be differentiated.

We used a single, multi-channel, continuous-wave NIRS system (Oxymon Mk III near-﻿infrared spectrophotometer; Artinis Medical Systems, Zetten, The Netherlands) as previously reported (Peltonen et al. 2013; Rissanen et al. 2015). The NIRS device used three simultaneously recording probes, each consisting of one receiving and three transmitting optodes operating at wavelengths of 765 and 860 nm, to detect changes from a set baseline. The Oxymon Mk III device also provides TSI% calculated from the light attenuation slope along the distance from the three emitting points as detected by the sensor of the receiving optode. The probes were placed over the VL muscle of the right leg at midthigh level and parallel to the long axis of the muscle, longitudinally on the BB muscle of the right arm above the elbow joint and lateral to the midline, and over the right frontal cortex, about 2 cm above the right eyebrow and as laterally as possible to the longitudinal cerebral fissure. Both hands were placed on the cycle handlebar and participants were instructed to keep their right hand relaxed and avoid a strong grip on the handlebar to minimize BB muscle activation during the test. The optodes were housed in an optically dense plastic holder, attached to the skin by a double-sided adhesive tape and covered by an elastic tape and a special headband was used for the cerebral probe. The inter-optode distances of the middle optode were chosen to be between 35 and 50 mm so that good signal quality was reached. The median distances used were 45, 42.5 and 50 mm for the leg, arm, and brain, respectively. The values used for the differential pathlength factor (DPF) were 5.51 for the leg, and 4.16 for the arm. DPF for cerebral tissue was calculated (DPF = 4.99 + 0.067 × Age^0.814^) according to the manufacturer’s guidelines.

Subcutaneous adipose tissue is an important source of error in NIRS measurements (Barstow 2019) and we therefore measured adipose tissue thickness (ATT) at the arm and thigh with a skinfold caliper. As the penetration depth of the NIRS signal is approximately half of the source-detector distance used, we set an ATT cutoff of 18 mm. Participants with ATT values exceeding this cutoff were excluded. In addition, we studied the NIRS measurements of each participant individually and data were deemed out of physiological range and excluded if it deviated grossly from previously reported patterns (Peltonen et al. 2013; Caen et al. 2019). Reasons for exclusion were VL HHb values not increasing, or a nonlinear response (excessive scattering) or a straight line (e.g. TSI constantly at 100%) for an extended period in any measurement.

### Vastus lateralis muscle blood flow

Ferreira et al. (2007) and Murias et al. (2013) developed a method to assess the profiles of regional VL blood flow (Q̇_VL_) as a function of V̇O_2_ from steady state to peak exercise. Extending their original approach, we adopted it to compare Q̇_VL_ between groups at different exercise intensities as previously reported (Rissanen et al. 2015). We calculated Q̇_VL_ using the formula

$$\dot{Q}_{VL} \, = \,\dot{V}O_{2} /C\left( {a - v} \right)O_{2VL,}$$

where V̇O_2_ is pulmonary oxygen uptake and C(a-v)O_2VL_ is the arteriovenous oxygen difference of VL. As the increase in global V̇O_2_ during cycling is mainly caused by increasing leg muscle oxygen uptake, 20 s left-shifted V̇O_2_ was used in the formula as a surrogate for local VL V̇O_2_. 20 s accounts for the circulatory transit delay from the lungs to the muscle (Murias et al. 2011, 2013). C(a-v)O_2VL_ was calculated with the formula

$$C\left( {a - v} \right)O_{2VL} = 10 + \left( {\% HHb/100\% } \right) \times 8,$$

where %HHb is the VL HHb signal from unloaded cycling to peak exercise normalized to 0–100%. 10 ml O_2_/100 ml blood represents the assumed muscle C(a-v)O_2_ at unloaded cycling and 8 ml O_2_/100 ml blood corresponds to the assumed change in C(a-v)O_2_ from unloaded cycling to peak exercise (Ferreira et al. 2007).

### Statistical analysis

We assessed data distribution with visual inspection and the Shapiro–Wilk test and either t-test or Mann–Whitney U test was used to analyze between group differences. We chose nonparametric statistical methods for the analyzes of the NIRS-results due to non-normal distribution of several variables. Q̇_VL_ was normally distributed, and regression models were used. Between-group differences (FM and CON) in O_2_Hb, HHb, tHb and TSI% at VL, BB and PFC at six time points (unloaded cycling (~ 6 W), 25 W, 50 W, 75 W, 100 W, and peak exercise) were studied. 100 W was the highest workload each participant could reach and was hence the last submaximal intensity for between-group comparisons. The mean of the last 30 s of each workload was calculated. Unloaded cycling was considered a steady state of tissue oxygenation and used as the zero reference point for comparison with subsequent results. Data were analyzed as concentration changes (μM) relative to unloaded cycling, as previously reported (Peltonen et al. 2013). We adjusted P-values with the Benjamini–Hochberg method for each NIRS variable separately, such as VL HHb from 6 W to peak exercise, to decrease the risk of type I error associated with multiple comparisons. To account for the difference in maximal exercise intensity, all variables were additionally analyzed at exercise intensities relative to V̇O_2peak_ using individually interpolated values to align with the corresponding V̇O_2_. 25% of V̇O_2peak_ approximated unloaded cycling and was included only in the Q̇_VL_ figure for illustrative purposes. Within-group differences in the NIRS variables from unloaded cycling to peak exercise were assessed using Friedman’s test followed by Dunn’s post hoc test. We compared peak exercise NIRS responses between VL and BB with Mann–Whitney U test, separately for FM and CON. Alpha was set to 0.05.

We constructed four different regression models to evaluate the relationship between local Q̇_VL_ and changes in factors (V̇O_2_, Q̇, MAP and SVR) affecting blood flow at a systemic level. The rationale for these analyzes was to investigate whether possible between-group differences in Q̇_VL_ would be independent of or related to systemic factors. Q̇_VL_ was the dependent variable and V̇O_2_, Q̇, MAP and SVR the independent variable, respectively. Group means from six time points (work-rates from 6 W to peak exercise) were used. Visual inspection of the scatter plots indicated a quadratic relationship between Q̇_VL_ and the independent variables. A nonlinear curve of leg blood flow to increasing exercise intensity is supported by previous literature (Calbet et al. 2007; Ferreira et al. 2007). Thus, a second-degree equation was formulated:

$$y\, = \,b_{1} \, + \,b_{2} x\, + \,b_{3} x^{2} ,$$

where y = Q̇_VL_, x = independent variable. To study the difference between FM and the control group we utilized dummy coding, which allows categorical variables to be included in linear regression models (Lunt 2015). A dummy variable named FM was added to the equation where patients got the value of 1 and the controls 0. Interactions between the dummy variable and the independent variable (FM*x, FM*x^2^) were introduced and the final model was constructed:

$$y\, = \,b_{1} \, + \,b_{2} x\, + \,b_{3} x^{2} \, + \,b_{4} FM\, + \,b_{5} FM*x\, + \,b_{6} FM*x^{2}$$

As there is an underlying physiological rationale for the use of the quadratic model and it was predetermined that between-group differences would be studied with the dummy variable and its interactions, we used the complete model as above, regardless of the P-values of individual terms.

Statistical analyses were conducted using SPSS (IBM SPSS Statistics for Windows, version 28.0 Armonk, NY, USA).

## Results

Participants were excluded due to technical issues, such as loss of NIRS signal quality (FM: n = 8, CON: n = 1), excess ATT (FM n = 5, CON n = 6), and data being out of physiological range (FM n = 9, CON n = 3). One participant in the CON group could not complete the exercise test. Ultimately, data was available for 16 FM patients and 17 CON participants. Further exclusions were made for specific analyses due to non-physiological NIRS responses (BB: 1 FM, 2 CON; PFC: 2 FM, 2 CON).

### Baseline characteristics and exercise outcomes

The groups were comparable in terms of age, body mass index (BMI), body fat percentage, ATT, and smoking status (Table 1). Patients with FM had significantly lower V̇O_2,_ work-rate, and systemic C(a-v)O_2_ at peak exercise. Peak Q̇, RER, SVR, MAP, and PETCO_2_ were not significantly different between the groups.

### NIRS and hemodynamic responses

**Fig. 2:**
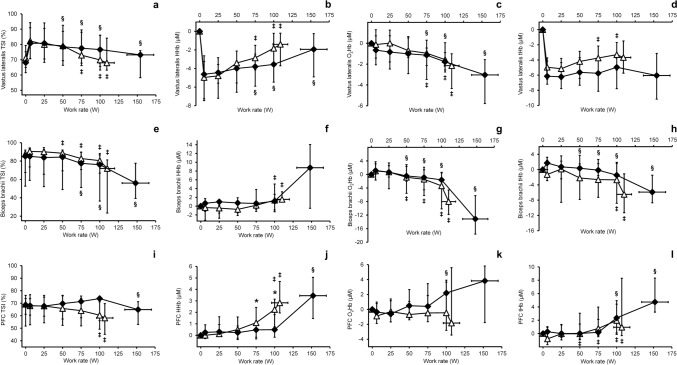
Oxygenation profiles in vastus lateralis (**a**–**d**), biceps brachii (**e**–**h**) and prefrontal cortex of the brain (PFC) (**i**–**l**) during incremental exercise. *TSI %* tissue saturation index, *HHb* deoxygenated hemoglobin, *O*_*2*_*Hb* oxygenated hemoglobin, *tHb* total hemoglobin. White triangles, fibromyalgia; black diamonds, controls. Datapoints represent group median and vertical error bars indicate interquartile range. Horizontal error bars illustrate standard deviation of peak work rate. *, change from unloaded cycling significantly different between groups. ‡, significant change from unloaded cycling in the fibromyalgia group; § significant change from unloaded cycling in the control group

**Fig. 3:**
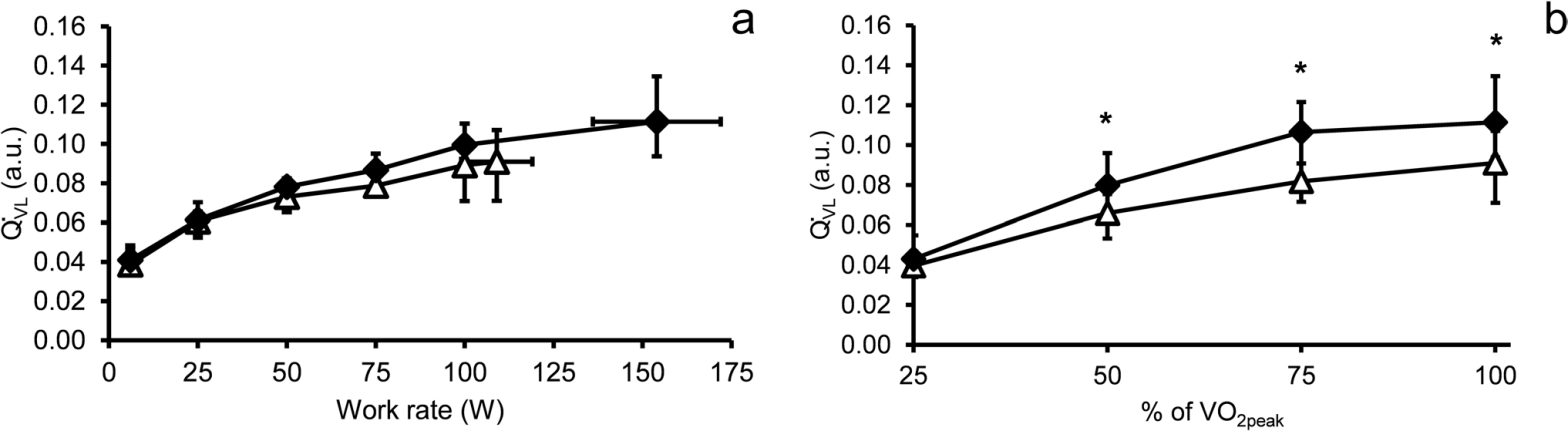
Vastus lateralis blood flow (Q̇_VL_) at absolute workloads (**a**) and relative to peak oxygen uptake (**b**). White triangles, patients with fibromyalgia; black diamonds, controls. Each datapoint represents group median and vertical error bars represent interquartile range. Horizontal error bars illustrate standard deviation of peak work rate. *, *P* < 0.05

NIRS results are illustrated in Fig. 2 and Supplementary Tables 1 and 2 (Online resource). No statistically significant between-group differences emerged in VL or BB NIRS variables at either absolute or relative workloads. In both groups, BB compared with VL showed greater decreases in peak exercise TSI% (FM: −16.64 [9.27] vs. −9.07 [11.11] percentage points, *P* = 0.036; CON: −29.60 [28.65] vs. −10.45 [10.18] percentage points, *P* = 0.020), O_2_Hb (FM: −6.38 [6.18] vs. −2.04 [3.83] μM, *P* < 0.001; CON: −14.04 [9.22] vs. −2.63 [3.50] μM, *P* < 0.001), and tHb (FM: −3.02 [6.47] vs. 0.83 [3.72] μM, *P* < 0.001; CON: −6.60 [5.30] vs. −0.27 [4.35] μM, *P* < 0.001). Peak HHb in BB compared with VL was not statistically different (FM: 2.77 [4.75] vs. 1.87 [3.98] μM, *P* = 0.813; CON: 7.94 [16.84] vs. 1.82 [3.59] μM, *P* = 0.193). PFC HHb was higher in the FM group at 75 W (1.09 [2.23] vs. −0.18 [1.38] μM, *P* = 0.033) and 100 W (2.20 [2.96] vs. 0.06 [1.92] μM, *P* = 0.005), but no difference was observed at relative workloads. No significant between-group differences emerged in PFC TSI%, O_2_Hb or tHb. Q̇_VL_ responses are presented in Fig. 3 and Table 2. Q̇_VL_ was similar between groups at absolute workloads, but lower in the FM group at 50%, 75%, and 100% of V̇O_2peak_.

**Table 2:** Vastus lateralis blood flow (Q̇_VL_)

	Fibromyalgia (*n* = 16)	Controls (*n* = 17)	*P*^‡^
	Median [IQR]	Median [IQR]	
Absolute workloads			
6 W	0.039 [0.033 to 0.047]	0.041 [0.035 to 0.048]	0.800
25 W	0.061 [0.052 to 0.070]	0.061 [0.055 to 0.066]	0.829
50 W	0.073 [0.065 to 0.082]	0.078 [0.072 to 0.081]	0.515
75 W	0.079 [0.077 to 0.089]	0.087 [0.076 to 0.095]	0.428
100 W	0.089 [0.071 to 0.096]	0.099 [0.086 to 0.110]	0.240
Relative to V̇O_2peak_			
25%	0.040 [0.034 to 0.047]	0.043 [0.037 to 0.055]	0.179
50%	0.066 [0.053 to 0.075]	0.080 [0.068 to 0.096]	0.013*
75%	0.082 [0.071 to 0.091]	0.106 [0.083 to 0.122]	0.013*
100%	0.091 [0.071 to 0.107]	0.111 [0.094 to 0.135]	0.013*

All data are in arbitrary units.* P*-values refer to Mann–Whitney U test. ‡, Benjamini–Hochberg adjustment for multiple comparisons. *, *P* < 0.05

### Regression analysis and model fit evaluation

**Fig. 4:**
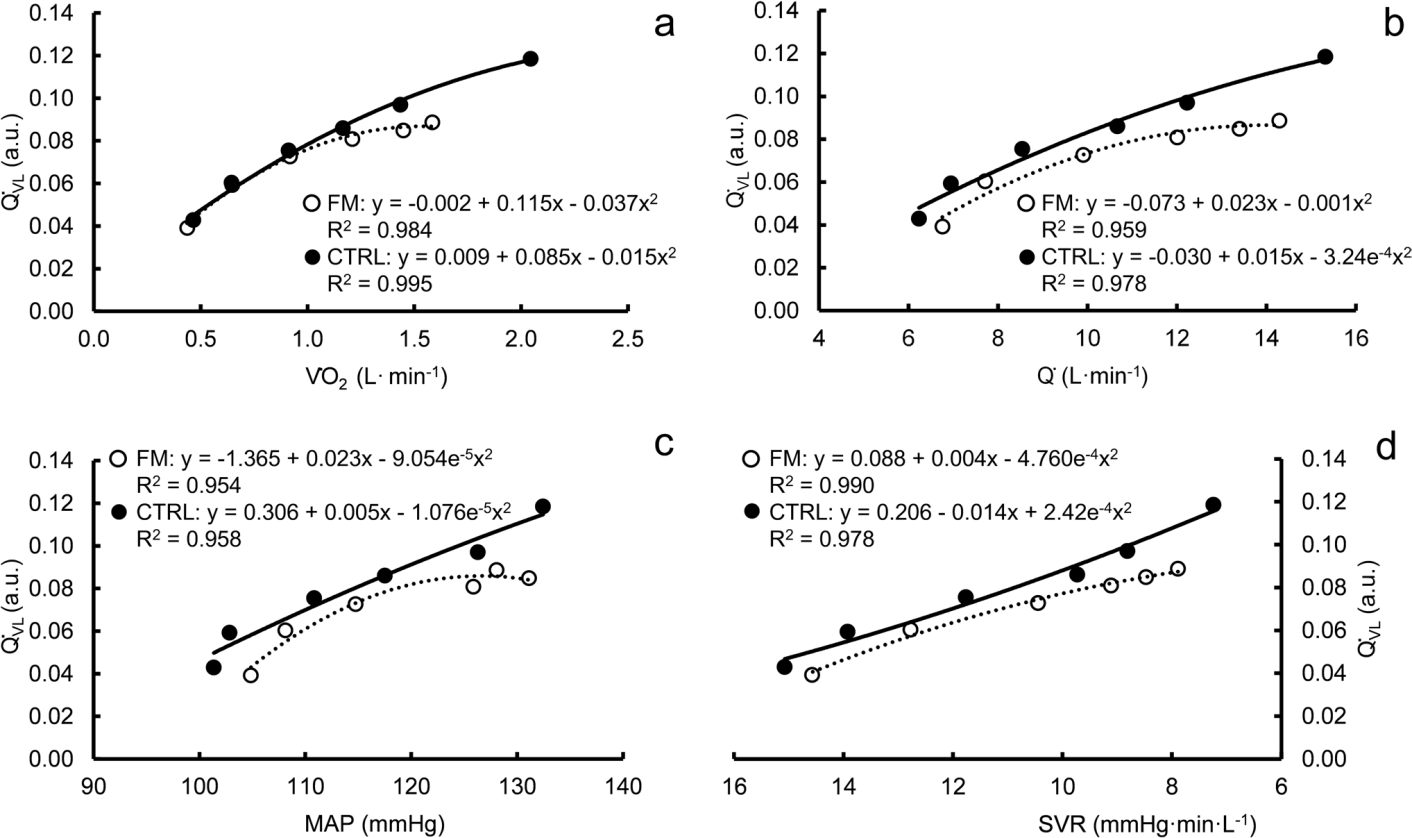
Vastus lateralis blood flow (Q̇_VL_) as a function of oxygen uptake (V̇O_2_) (**a**), cardiac output (Q̇) (**b**), mean arterial pressure (MAP) (**c**) and systemic vascular resistance (SVR) (**d**). White circles and dotted line, fibromyalgia; black circles and solid line, controls. Each datapoint represents group mean. *A.u.* arbitrary units

The regression curves are depicted in Fig. 4. The step-by-step construction of the models and the corresponding fit of the model are presented in Online resource, Supplementary Table 3. The initial models with only the independent variables and their squares were statistically significant (Q̇_VL_ vs. V̇O_2_, R^2^ = 0.938, *P* < 0.001; Q̇_VL_ vs. Q̇, R^2^ = 0.839, *P* < 0.001; Q̇_VL_ vs. MAP, R^2^ = 0.792, *P* < 0.001; Q̇_VL_ vs. SVR, R^2^ = 0.876, *P* < 0.001). Adding the dummy variable FM increased the F-values significantly in the models with Q̇ (ΔR^*2*^ = 0.108, ΔF = 16.4, *P* = 0.004), MAP (ΔR^*2*^ = 0.139, ΔF = 16.0, *P* = 0.004) and SVR (ΔR^*2*^ = 0.090, ΔF = 20.8, *P* = 0.002), but not with V̇O_2_ (ΔR^*2*^ = 0.023, ΔF = 4.9, *P* = 0.058). Adding the interaction term FM*independent variable increased the fit of the model significantly only in the case of V̇O_2_ (ΔR^*2*^ = 0.024, ΔF = 11.4, *P* = 0.012). Adding the second-degree interaction terms did not improve any of the models significantly, although the complete models reached high correlations (adjusted R^2^ = 0.985; 0.951; 0.909 and 0.968 in Q̇_VL_ vs. V̇O_2,_ Q̇_VL_ vs. Q̇, Q̇_VL_ vs. MAP and Q̇_VL_ vs. SVR, respectively). Statistically significant coefficients of individual terms in the complete models (Online resource, Supplementary Table 4) were V̇O_2_ (*P* < 0.001), V̇O_2_ squared (*P* = 0.012), and Q̇ (*P* = 0.030), whereas the dummy variable FM or its interaction terms were not statistically significant in any of the complete models.

## Discussion

In this study, we measured the (de)oxygenation responses of the active leg muscle, the relatively inactive arm muscle, and the prefrontal cerebral cortex during incremental cycling in patients with FM compared with healthy controls. Our key findings were (1) similar VL oxygenation between groups, (2) lower Q̇_VL_ at 50%, 75%, and 100% of V̇O_2peak_, but not at absolute workloads, and (3) higher PFC HHb at 75 W and 100 W, but not at relative workloads, in the FM group.

### Muscle oxygenation during exercise in fibromyalgia

In healthy subjects, VL HHb and tHb increase whereas O_2_Hb and TSI% decrease during incremental cycling suggesting increased oxygen extraction in the exercising muscle (Orcioli-Silva et al. 2024). The capability to extract oxygen correlates with fitness and is enhanced by exercise training, where subjects with higher V̇O2_peak_ have greater amplitudes of deoxygenation (Boone et al. 2016; Okushima et al. 2016; Caen et al. 2019). In line with our hypothesis, patients with FM and healthy controls showed similar responses in VL HHb and O_2_Hb at submaximal workloads. However, contrary to our second hypothesis, no differences were observed at peak exercise. Estimated Q̇_VL_ was lower in FM subjects at peak exercise and at submaximal exercise relative to V̇O_2peak_ but no significant between group differences were observed at absolute workloads, although mean Q̇_VL_ was 6 to 10% lower at 50 to 100 W in the FM group. These results suggest that muscle blood flow is coupled with muscle metabolic rate irrespective of V̇O_2peak_ and this coupling is not impaired in FM. In addition, Q̇_VL_ in relation to increasing V̇O_2_, Q̇, MAP, and decreasing SVR exhibited visible differences between the groups implying that the difference is not related to systemic, but to local blood flow regulation and the metabolic demand of the working muscle. However, we were unable to confirm a significant difference, as the coefficients of the dummy variables and interaction terms were not statistically significant in the regression analysis. Whether decreased peak Q̇_VL_ limits exercise capacity is beyond the scope of the study design, but compromised oxygen delivery would be expected to result in pronounced increases in deoxygenation and decreases in O_2_Hb and TSI%, which were not observed. Thus, reduced exercise capacity in FM does not seem to be associated with muscle oxygenation. It should be noted that interpreting the results of a single NIRS probe warrants caution (Barstow 2019) as heterogeneity in deoxygenation (Koga et al. 2011) and blood flow (Vogiatzis et al. 2015) in the quadriceps femoris muscle is evident. However, vastus lateralis blood flow measured by NIRS using indocyanine green (Vogiatzis et al. 2009) yields results comparable to those obtained from invasive femoral artery blood flow measurements (Mortensen et al. 2008).

There was no difference in VL peak HHb although the two groups were markedly different regarding their fitness status measured by V̇O_2peak_. This could be explained by subject characteristics as the subjects in previous studies demonstrating a correlation between V̇O2_peak_ and HHb amplitude consisted of young men with considerably higher V̇O_2peak_ (Boone et al. 2016; Okushima et al. 2016; Caen et al. 2019)_._ The discrepancy between reduced peak exercise C(a-v)O_2_ in the FM group but similar HHb and TSI% between groups imply that regionally measured oxygenation is not sensitive enough to detect changes in whole body C(a-v)O_2_ (Bhambhani et al. 1999) and is likely associated with different regulatory mechanisms behind systemic and active muscle microvascular perfusion and oxygen extraction (Murias et al. 2013).

Oxygenation responses of inactive BB during incremental cycling are less established, but a previous study (Peltonen et al. 2013) showed a pattern of increasing HHb and decreasing TSI%. These are also documented during incremental treadmill exercise (Rissanen et al. 2012), although the extent of BB activation during treadmill exercise and cycling are likely different. Greater TSI% response in the arm compared to the leg observed in this current study aligns with previous reports from our laboratory using cycling (Peltonen et al. 2012, 2013) and treadmill protocols (Rissanen et al. 2012), but contrasts other studies using invasive venous oxygen measurements (Calbet et al. 2007, 2015). The reason for this discrepancy remains unclear. Nevertheless, greater desaturation in the arm might reflect redirection of cardiac output from inactive muscles to the active leg muscles. The exercise pressor reflex and central command increase sympathetic and decrease parasympathetic activity, leading to vasoconstriction, while functional sympatholysis and vasodilatation are enhanced in the contracting muscles (Grotle et al. 2020). This could result in less blood flow, decreased oxygen availability, and increased deoxygenation in the arm. Supporting this, the BB compared to VL showed lower TSI% and O_2_Hb, higher HHb, but lower total Hb at peak exercise (Online resource, Supplementary Table 1).

Moreover, lower BB TSI%, O_2_Hb, tHb, and higher HHb in the controls at peak exercise (Online resource, Supplementary Table 1), although not statistically significant, suggest that the control group displayed more extensive blood flow redistribution away from the arm at peak exercise. This potential difference in circulatory regulation could be related to lower achieved peak V̇O_2_ and work-rate in the FM group. However, it has been shown that patients with FM have reduced chronotropic responses to exercise irrespective of their fitness (Schamne et al. 2021), as well as blunted heart rate variability responses to cognitive tasks (Zetterman et al. 2023), indicating attenuated sympathetic responses to stress which could reduce exercise induced vasoconstriction.

Previous reports studying muscle oxygenation in FM had different protocols than our current study. Some similarities can however be found. Dinler et al. (2007) initially reported slower oxygen kinetics in FM patients compared with controls using a forearm ischemia protocol, but the difference was not significant in their later study (Dinler et al. 2009). Shang et al. (2012) used a similar protocol and an isometric leg exercise protocol and found again slower oxygen recovery in FM patients. Additionally, Shang et al. reported lower oxygen extraction, but similar blood flow, in FM patients compared with controls. The conclusion of lower oxygen extraction is based on a single parameter, relative oxygen extraction fraction, while there was no difference in the conventional NIRS parameters: oxy-, deoxy, and total hemoglobin, or oxygen saturation. The authors of the study provide no explanation for this discrepancy (Shang et al. 2012). In a study by Srikuea et al. (2013), lower capillary density, but no difference in muscle oxygenation was found between FM patients and controls. On the contrary, Villafaina et al. (2023), observed altered muscle (VL) oxygenation in FM patients during fatiguing leg exercise. The patients and controls in their study were markedly different regarding BMI and although the authors used BMI as a covariate in their analyzes, the presumably higher subcutaneous adiposity in the patient group questions the reliability of these NIRS-derived results. Schamne et al. (2024) examined oxygen saturation in the VL muscle during constant cycling and found no differences between FM and controls. It should be noted that none of the afore mentioned studies controlled for adipose tissue thickness. In conclusion, our results of similar muscle oxygenation between patients and controls are in concordance with most previous studies (Shang et al. 2012; Srikuea et al. 2013; Schamne et al. 2024).

To our knowledge, there are two previous studies that have examined muscle blood flow during exercise in FM. Elvin et al. (2006) studied the infraspinatus muscle during dynamic exercise with contrast-enhanced ultrasound and found decreased blood flow in FM patients. Their protocol consisted of outward rotations of the shoulder against a resistance band in a normal chair, which is in many regards an uncontrolled setting and minuscule differences in effort might have influenced the results. The previously mentioned study by Shang et al. (2012) used diffuse correlation spectroscopy to assess blood flow of the knee extensor muscles during isometric contractions and found no difference between FM patients and controls. In comparison to our finding of reduced peak exercise Q̇_VL_ in FM, a single leg exercise protocol might not be sufficient to elicit differences in blood flow between FM and controls whereas cycling poses more demands to the cardiovascular system and blood flow distribution to the exercising muscles.

### Cerebral oxygenation during exercise in fibromyalgia

Previous studies have reported PFC HHb to increase and TSI% to decrease towards maximal exercise in healthy subjects (De Wachter et al. 2021; Orcioli-Silva et al. 2024). PFC O_2_Hb has been reported to rise until the respiratory compensation point, but studies are not consistent regarding O_2_Hb responses at maximal exercise (De Wachter et al. 2021). In the present study, a pattern of rising O_2_Hb was observed in the CON but was not as evident in the FM group. HHb increased towards peak exercise in both groups but was higher at 75 W and 100 W in the FM group. Peak exercise PFC O_2_Hb and tHb appeared lower in the FM group, which implies lower blood volume. Cerebral blood flow during exercise is regulated by multiple factors, including the partial pressures of arterial carbon dioxide (PaCO_2_) and oxygen (PaO_2_), Q̇, cerebral metabolism, blood pressure, and neural regulation (Smith and Ainslie 2017). PETCO_2_, a surrogate for PaCO_2_, and MAP were similar between groups at peak exercise offering no explanation for the possible difference in PFC O_2_Hb and tHb. Q̇ was slightly, but non-significantly lower in the FM group, but the influence of Q̇ on cerebral blood flow is likely modest (Smith and Ainslie 2017) and not consistently observed under all conditions (Watanabe et al. 2024). In addition, both groups terminated the exercise at similar PFC deoxygenation (HHb) and saturation (TSI%) levels indicating that the mismatch between oxygen supply and demand at peak exercise was similar between groups. Faster deoxygenation at submaximal, but similar at peak exercise, and a blunted oxygenation response might reflect slower adaptation of cerebral blood flow and greater initial reliance on oxygen extraction in FM.

### Methodological strengths and limitations

One of the key strengths of our study is the rigorous evaluation and exclusion of NIRS data that did not meet physiological criteria, ensuring that our results reflect true muscle and cerebral oxygenation rather than artifacts caused by subcutaneous adipose tissue or technical issues. Additionally, the use of multiple probes allows for a comprehensive assessment of both peripheral and cerebral oxygenation simultaneously. However, several limitations must be acknowledged. A recent study has questioned the reliability of NIRS measurements during exercise in subjects with ATT > 8 mm (Stuer et al. 2024). There are two important methodological differences compared to our current study: 1) using a frequency domain vs. constant wave NIRS and 2) studying constant cycling vs. incremental exercise. Moreover, the authors speculate whether the differences between men and women are truly attributable to ATT or other physiological differences between sexes. Although higher ATT in our study might have diminished the amplitudes of NIRS results, we did observe physiologically logical patterns that are comparable to previous studies. Low sample size combined with large interindividual differences in oxygenation responses, which are common in NIRS studies, makes it challenging to accurately detect between-group differences. A quadratic regression model with a dummy variable and interaction terms increases collinearity and inflates p-values for individual terms, raising the risk of type II error. Furthermore, excluding subjects with higher ATT limits the generalizability of our results. Our method to calculate Q̇_VL_ has not been validated and provides only a tentative estimate of vastus lateralis blood flow. Moreover, this approach was initially developed to compare the kinetics of Q̇_VL_ (i.e. the shape of the curve) as a function of exercise intensity (Ferreira et al. 2007; Murias et al. 2013), but not its absolute values (i.e. the height of the curve) between study groups. While this method has been used to compare Q̇_VL_ between diabetic patients and healthy controls (Rissanen et al. 2015), its applicability remains limited, particularly in the absence of ischemic calibrations. We did not perform ischemic calibrations to determine reference values for peak HHb, and uncertainty exists whether the values obtained during peak exercise accurately reflect true maximal deoxygenation in all subjects. The assumed C(a-v)O_2_ values for unloaded cycling (10 ml O_2_/100 ml blood) and peak exercise (18 ml O_2_/100 ml blood), as well as the 20 s transit time from lungs to the exercising muscle, may not be equally applicable to patients with FM and healthy controls. Specifically, if a deficit in oxidative metabolism was present in FM, venous O_2_ concentration would remain higher and muscle C(a-v)O_2_ lower, leading to an underestimation of Q̇_VL_ in the FM group. However, the similar vastus lateralis TSI% and HHb responses in both groups suggest no clear evidence of impaired oxygen utilization in the FM group. Finally, our study population consisted of women only and the results cannot be extrapolated to male FM patients.

## Conclusions

Oxygenation profiles of exercising muscle and PFC deoxygenation at peak exercise did not differ between patients with FM and healthy controls. This study adds to the evidence that exercise intolerance in FM may not be related to muscle abnormalities, and the deviations observed might reflect deconditioning and lower cardiorespiratory fitness. Further studies are however needed to provide additional insights on the role of cerebral oxygenation and blood flow distribution during exercise, as well as other mechanistic pathways that may lead to pain, fatigue and exercise intolerance in individuals suffering from FM.

## Supplementary Information

Below is the link to the electronic supplementary material.

Supplementary file1 (DOCX 46 KB)

## Data Availability

The datasets generated and analyzed during the current study are not publicly available as consent for this was not asked from the study subjects. The data are available from the corresponding author on reasonable request if also approved by our ethics committee.

## Abbreviations

%HHb: Normalized deoxyhemoglobin
ATT: Adipose tissue thickness
BB: Biceps brachii
C(a-v)O_2_: Arteriovenous oxygen difference
C(a-v)O_2VL_: Arteriovenous oxygen difference in vastus lateralis
CON: Control group
DPF: Differential pathlength factor
FIQ: Fibromyalgia impact questionnaire
FM: Fibromyalgia
HHb: Deoxyhemoglobin
ICG: Impedance cardiography
MAP: Mean arterial pressure
NIRS: Near-infrared spectroscopy
O_2_Hb: Oxyhemoglobin
PaCO_2_: Partial pressure of arterial carbon dioxide
PETCO_2_: Partial pressure of end-tidal carbon dioxide
PFC: Prefrontal cortex
Q̇: Cardiac output
Q̇_VL_: Vastus lateralis blood flow
RER: Respiratory exchange ratio
SVR: Systemic vascular resistance
tHb: Total hemoglobin
TSI%: Tissue saturation index
V̇CO_2_: Carbon dioxide production
V̇E: Minute ventilation
VL: Vastus lateralis
V̇O_2_: Oxygen uptake
V̇O_2peak_: Peak oxygen uptake
