# Supplementary material for: Muscle and cerebral oxygenation during exercise in fibromyalgia: a near-infrared spectroscopy study
Source: Eur J Appl Physiol. 2025 Oct 9;126(3):1723–35. doi: 10.1007/s00421-025-06013-8 (PMC13013206; doi:10.1007/s00421-025-06013-8)
Supplement: Supplementary file 1 — Supplementary file1 (DOCX 46 KB) [file 421_2025_6013_MOESM1_ESM.docx]

**Muscle and Cerebral Oxygenation During Exercise in Fibromyalgia: A Near-infrared Spectroscopy Study**

Taneli Lehto^1^, Teemu Zetterman, Dominique Gagnon, Ritva Markkula, Jari Arokoski, Eija Kalso, and Juha E. Peltonen

1. Sports and Exercise Medicine, Faculty of Medicine, University of Helsinki, Helsinki, Finland

Address for correspondence: Taneli Lehto, MD, Sports and Exercise Medicine, Faculty of Medicine, University of Helsinki, Mäkelänkatu 47, Urhea-hall, 00550 Helsinki, Finland. E-mail: [taneli.lehto@helsinki.fi](mailto:taneli.lehto@helsinki.fi).

| SUPPLEMENTARY TABLE 1. Change in NIRS variables relative to unloaded cycling. | | | | |  |
| --- | --- | --- | --- | --- | --- |
|  | **Fibromyalgia** | | **Controls** | |  |
|  | **n** | **Median [IQR]** | **n** | **Median [IQR]** | ***P*^‡^** |
| Vastus lateralis | 16 |  | 17 |  |  |
| TSI 25 W |  | -0.42 [-2.39 to 0.73] |  | -0.54 [-1.37 to 0.23] | 0.943 |
| TSI 50 W |  | -1.96 [-4.87 to -0.63] |  | -1.89 [-2.88 to -0.50] | 0.933 |
| TSI 75 W |  | -4.63 [-6.86 to -2.31] |  | -3.36 [-4.93 to -1.22] | 0.518 |
| TSI 100 W |  | -7.57 [-10.32 to -3.78] |  | -5.11 [-7.61 to -2.57] | 0.450 |
| TSI peak |  | -9.07 [-15.57 to -4.46] |  | -10.45 [-15.74 to -5.56] | 0.933 |
| O_2_Hb 25 W |  | -0.16 [-0.71 to 0.02] |  | -0.09 [-0.78 to 0.24] | 0.900 |
| O_2_Hb 50 W |  | -0.34 [-1.21 to 0.29] |  | -0.41 [-1.12 to 0.10] | 1.000 |
| O_2_Hb 75 W |  | -0.98 [-1.62 to 0.00] |  | -0.86 [-2.05 to -0.14] | 1.000 |
| O_2_Hb 100 W |  | -1.66 [-3.09 to -0.50] |  | -1.36 [-2.04 to 0.13] | 0.828 |
| O_2_Hb peak |  | -2.04 [-4.35 to -0.52] |  | -2.63 [-4.87 to -1.37] | 0.828 |
| HHb 25 W |  | -0.02 [-0.40 to 0.80] |  | -0.10 [-0.19 to 0.21] | 0.640 |
| HHb 50 W |  | 0.49 [-0.02 to 2.20] |  | 0.39 [-0.10 to 0.74] | 0.484 |
| HHb 75 W |  | 1.02 [0.50 to 3.69] |  | 0.75 [0.20 to 1.47] | 0.484 |
| HHb 100 W |  | 1.68 [0.79 to 4.91] |  | 1.05 [0.35 to 2.16] | 0.420 |
| HHb peak |  | 1.87 [1.28 to 5.26] |  | 1.82 [1.08 to 4.67] | 0.484 |
| tHb 25 W |  | -0.08 [-0.36 to 0.13] |  | -0.18 [-0.65 to 0.34] | 0.540 |
| tHb 50 W |  | 0.42 [-0.28 to 1.40] |  | 0.11 [-0.67 to 0.74] | 0.244 |
| tHb 75 W |  | 0.87 [-0.14 to 2.68] |  | 0.28 [-1.31 to 1.22] | 0.244 |
| tHb 100 W |  | 1.43 [-0.05 to 3.27] |  | 0.36 [-0.84 to 1.68] | 0.244 |
| tHb peak |  | 0.83 [-0.27 to 3.45] |  | -0.27 [-2.92 to 1.43] | 0.244 |
| Biceps brachii | 15 |  | 15 |  |  |
| TSI 25 W |  | -0.54 [-2.06 to 0.51] |  | -1.07 [-2.62 to 0.34] | 0.885 |
| TSI 50 W |  | -2.66 [-6.15 to -0.95] |  | -2.11 [-4.30 to -0.73] | 0.755 |
| TSI 75 W |  | -6.02 [-10.94 to -2.26] |  | -3.35 [-7.56 to -1.27] | 0.550 |
| TSI 100 W |  | -9.93 [-18.18 to -5.08] |  | -6.30 [-10.61 to -2.27] | 0.550 |
| TSI peak |  | -16.64 [-18.81 to -9.54] |  | -29.60 [-35.80 to -7.15] | 0.550 |
| O_2_Hb 25 W |  | -0.40 [-0.95 to 0.28] |  | -0.54 [-2.80 to -0.06] | 0.517 |
| O_2_Hb 50 W |  | -1.18 [-1.79 to -0.69] |  | -1.52 [-2.64 to -1.18] | 0.380 |
| O_2_Hb 75 W |  | -2.94 [-5.05 to -0.34] |  | -2.54 [-3.96 to -1.76] | 0.852 |
| O_2_Hb 100 W |  | -4.64 [-7.09 to -1.72] |  | -5.11 [-5.39 to -1.01] | 0.852 |
| O_2_Hb peak |  | -6.38 [-10.63 to -4.45] |  | -14.04 [-17.22 to -8.00] | 0.130 |
| HHb 25 W |  | -0.03 [-0.29 to 0.58] |  | 0.31 [-0.92 to 1.19] | 0.905 |
| HHb 50 W |  | 0.17 [-0.35 to 1.35] |  | 0.58 [-0.33 to 1.56] | 0.917 |
| HHb 75 W |  | 0.61 [0.02 to 3.57] |  | 0.78 [-0.21 to 3.11] | 0.905 |
| HHb 100 W |  | 2.07 [0.30 to 5.41] |  | 1.49 [-0.37 to 3.37] | 0.905 |
| HHb peak |  | 2.77 [1.30 to 6.05] |  | 7.94 [-1.12 to 15.72] | 0.905 |
| tHb 25 W |  | -0.42 [-0.79 to 0.36] |  | -0.78 [-1.58 to -0.15] | 0.380 |
| tHb 50 W |  | -0.94 [-1.99 to 0.52] |  | -1.32 [-1.90 to -0.96] | 0.395 |
| tHb 75 W |  | -1.78 [-3.21 to 0.31] |  | -1.50 [-2.37 to -0.70] | 0.917 |
| tHb 100 W |  | -2.53 [-4.29 to -0.24] |  | -2.91 [-3.82 to -0.52] | 0.917 |
| tHb peak |  | -3.02 [-7.44 to -0.97] |  | -6.60 [-8.31 to -3.01] | 0.380 |
| PFC | 14 |  | 15 |  |  |
| TSI 25 W |  | 0.02 [-0.54 to 0.68] |  | -0.24 [-0.84 to 0.30] | 0.479 |
| TSI 50 W |  | -0.71 [-3.42 to 0.65] |  | 0.34 [-0.61 to 2.39] | 0.148 |
| TSI 75 W |  | -1.49 [-6.69 to 1.12] |  | 1.94 [-2.95 to 3.77] | 0.148 |
| TSI 100 W |  | -3.28 [-11.12 to -0.04] |  | 1.66 [-3.00 to 4.21] | 0.148 |
| TSI peak |  | -7.02 [-13.47 to -0.06] |  | -3.58 [-13.33 to 1.64] | 0.827 |
| O_2_Hb 25 W |  | 0.20 [-0.03 to 0.94] |  | -0.15 [-0.27 to 0.39] | 0.115 |
| O_2_Hb 50 W |  | 0.33 [-0.03 to 1.15] |  | 0.44 [-0.49 to 1.13] | 0.727 |
| O_2_Hb 75 W |  | 0.51 [-0.47 to 1.62] |  | 0.75 [0.20 to 2.15] | 0.727 |
| O_2_Hb 100 W |  | 0.32 [-1.21 to 3.28] |  | 1.79 [0.24 to 3.81] | 0.727 |
| O_2_Hb peak |  | -1.08 [-1.85 to 5.65] |  | 3.48 [-1.48 to 5.33] | 0.727 |
| HHb 25 W |  | 0.35 [-0.13 to 0.66] |  | 0.03 [-0.12 to 0.13] | 0.258 |
| HHb 50 W |  | 0.32 [-0.08 to 1.04] |  | -0.16 [-0.54 to 0.18] | 0.060 |
| HHb 75 W |  | 1.09 [-0.11 to 2.12] |  | -0.18 [-0.80 to 0.58] | 0.033* |
| HHb 100 W |  | 2.20 [0.65 to 3.61] |  | 0.06 [-0.92 to 1.00] | 0.005* |
| HHb peak |  | 2.83 [1.65 to 4.33] |  | 2.24 [0.24 to 4.00] | 0.337 |
| tHb 25 W |  | 0.37 [0.07 to 1.68] |  | -0.04 [-0.25 to 0.25] | 0.090 |
| tHb 50 W |  | 0.67 [0.36 to 1.66] |  | 0.23 [-0.37 to 0.73] | 0.090 |
| tHb 75 W |  | 1.11 [0.91 to 2.37] |  | 0.65 [-0.16 to 1.83] | 0.135 |
| tHb 100 W |  | 2.44 [0.98 to 3.80] |  | 1.59 [0.52 to 3.28] | 0.513 |
| tHb peak |  | 1.73 [0.94 to 8.67] |  | 4.13 [2.29 to 6.59] | 0.513 |
| All data are in µM, except for TSI in percentage points. *P*-values refer to Mann-Whitney U test. ‡, Benjamini-Hochberg adjustment for multiple comparisons. *, *P* < 0.05. TSI, tissue saturation index; O_2_Hb, oxyhemoglobin; HHb, deoxyhemoglobin; tHb, total hemoglobin; PFC, prefrontal cortex. | | | | | |

| SUPPLEMENTARY TABLE 2. NIRS results at 50 and 75% of peak oxygen uptake. | | | | | |
| --- | --- | --- | --- | --- | --- |
|  | **Fibromyalgia** | | **Controls** | |  |
|  | **n** | **Median [IQR]** | **n** | **Median [IQR]** | ***P*^‡^** |
| Vastus lateralis | 16 |  | 17 |  |  |
| TSI 50% |  | -1.52 [-4.31 to 0.11] |  | -1.30 [-4.56 to -0.02] | 0.986 |
| TSI 75% |  | -4.52 [-8.62 to -1.82] |  | -5.02 [-9.30 to -1.26] | 0.986 |
| O_2_Hb 50% |  | -0.22 [-1.29 to 0.08] |  | -0.70 [-1.34 to 0.16] | 1.000 |
| O_2_Hb 75% |  | -0.92 [-2.55 to 0.05] |  | -1.11 [-2.28 to -0.08] | 1.000 |
| HHb 50% |  | 0.28 [-0.13 to 1.64] |  | 0.37 [-0.08 to 1.01] | 0.901 |
| HHb 75% |  | 0.72 [0.54 to 3.29] |  | 1.06 [0.24 to 2.76] | 0.901 |
| tHb 50% |  | 0.33 [-0.32 to 0.95] |  | 0.22 [-0.82 to 0.87] | 0.709 |
| tHb 75% |  | 0.60 [-0.45 to 2.51] |  | 0.70 [-0.59 to 1.66] | 0.709 |
| Biceps brachii | 15 |  | 15 |  |  |
| TSI 50% |  | -1.38 [-2.85 to -0.24] |  | -1.37 [-3.63 to -0.83] | 0.624 |
| TSI 75% |  | -5.77 [-8.28 to -2.42] |  | -7.99 [-11.33 to -2.67] | 0.466 |
| O_2_Hb 50% |  | -0.73 [-1.56 to -0.09] |  | -1.46 [-2.52 to -0.52] | 0.174 |
| O_2_Hb 75% |  | -1.86 [-4.11 to -0.58] |  | -2.41 [-5.67 to -1.83] | 0.174 |
| HHb 50% |  | -0.05 [-0.21 to 0.68] |  | 0.45 [0.11 to 1.24] | 0.325 |
| HHb 75% |  | 0.31 [-0.16 to 2.39] |  | 2.33 [-0.25 to 4.35] | 0.325 |
| tHb 50% |  | -0.29 [-1.21 to 0.85] |  | -0.96 [-2.04 to -0.42] | 0.500 |
| tHb 75% |  | -1.86 [-2.64 to 0.24] |  | -2.40 [-3.09 to -0.93] | 0.512 |
| PFC | 14 |  | 15 |  |  |
| TSI 50% |  | -0.47 [-0.81 to 0.44] |  | 1.42 [-0.29 to 3.04] | 0.170 |
| TSI 75% |  | -0.61 [-3.66 to 1.11] |  | 0.02 [-3.47 to 7.44] | 0.400 |
| O_2_Hb 50% |  | 0.39 [0.02 to 0.87] |  | 0.54 [-0.12 to 1.94] | 0.477 |
| O_2_Hb 75% |  | 0.64 [-0.06 to 2.26] |  | 1.78 [0.68 to 4.07] | 0.477 |
| HHb 50% |  | 0.25 [-0.12 to 0.43] |  | -0.14 [-0.64 to 0.23] | 0.074 |
| HHb 75% |  | 0.60 [0.08 to 1.35] |  | 0.13 [-0.89 to 0.99] | 0.186 |
| tHb 50% |  | 0.40 [0.15 to 1.50] |  | 0.68 [-0.10 to 1.52] | 0.949 |
| tHb 75% |  | 1.57 [0.71 to 2.39] |  | 2.99 [-0.17 to 4.00] | 0.949 |
| Values reported as change (µM, except for TSI, percentage points) from unloaded cycling (corresponding to ~25% of peak oxygen uptake). *P*-values refer to Mann-Whitney U test. ‡, Benjamini-Hochberg adjustment for multiple comparisons. TSI, tissue saturation index; O_2_Hb, oxyhemoglobin; HHb, deoxyhemoglobin; tHb, total hemoglobin; PFC, prefrontal cortex. | | | | | |

| SUPPLEMENTARY TABLE 3. Step by step construction of regression models and fit of the models at each step. | | | | | | | |
| --- | --- | --- | --- | --- | --- | --- | --- |
| **Model predictors** | **R** | **R^2^** | **Adjusted R^2^** | **SE** | **R^2^ Change** | **F Change** | ***P*** |
| Q̇_VL_ vs V̇O_2_ |  |  |  |  |  |  |  |
| V̇O_2,_ V̇O_2_^^2^ | 0.969 | 0.938 | 0.924 | 0.006 | 0.938 | 68.334 | < 0.001* |
| V̇O_2,_ V̇O_2_^^2^, FM | 0.981 | 0.962 | 0.947 | 0.005 | 0.023 | 4.870 | 0.058 |
| V̇O_2,_ V̇O_2_^^2^, FM, FM*V̇O_2_ | 0.993 | 0.985 | 0.977 | 0.003 | 0.024 | 11.381 | 0.012* |
| V̇O_2,_ V̇O_2_^^2^, FM, FM*V̇O_2,_ FM*V̇O_2_^^2^ | 0.996 | 0.992 | 0.985 | 0.003 | 0.007 | 5.048 | 0.066 |
| Q̇_VL_ vs Q̇ |  |  |  |  |  |  |  |
| Q̇, Q̇^2^ | 0.916 | 0.839 | 0.803 | 0.010 | 0.839 | 23.450 | < 0.001* |
| Q̇, Q̇^2^, FM | 0.973 | 0.947 | 0.928 | 0.006 | 0.108 | 16.447 | 0.004* |
| Q̇, Q̇^2^, FM, FM*Q̇ | 0.984 | 0.968 | 0.950 | 0.005 | 0.021 | 4.496 | 0.072 |
| Q̇, Q̇^2^, FM, FM*Q̇, FM*Q̇^2^ | 0.986 | 0.973 | 0.951 | 0.005 | 0.005 | 1.158 | 0.323 |
| Q̇_VL_ vs MAP^a^ |  |  |  |  |  |  |  |
| MAP, MAP^2^ | 0.890 | 0.792 | 0.745 | 0.011 | 0.792 | 17.092 | < 0.001* |
| MAP, MAP^2^, FM | 0.965 | 0.930 | 0.904 | 0.007 | 0.139 | 15.964 | 0.004* |
| MAP, MAP^2^, FM, FM*MAP | 0.970 | 0.942 | 0.909 | 0.007 | 0.011 | 1.365 | 0.281 |
| Q̇_VL_ vs SVR |  |  |  |  |  |  |  |
| SVR, SVR^2^ | 0.936 | 0.876 | 0.848 | 0.009 | 0.876 | 31.688 | < 0.001* |
| SVR, SVR^2^, FM | 0.983 | 0.965 | 0.952 | 0.005 | 0.090 | 20.769 | 0.002* |
| SVR, SVR^2^, FM, FM*SVR | 0.988 | 0.977 | 0.963 | 0.004 | 0.011 | 3.340 | 0.110 |
| SVR, SVR^2^, FM, FM*SVR, FM*SVR^2^ | 0.991 | 0.983 | 0.968 | 0.004 | 0.006 | 2.160 | 0.192 |
| Q̇_VL_, vastus lateralis blood flow; V̇O_2_, oxygen uptake; FM, dummy variable (patients = 1, controls = 0); Q̇, cardiac output; MAP, mean arterial pressure; SVR systemic vascular resistance. | | | | | | | |

| SUPPLEMENTARY TABLE 4. Significance of individual coefficients in the regression models. | | | | | | |
| --- | --- | --- | --- | --- | --- | --- |
|  | **β** | **SE** | **95% CI** | **Standardized β** | **t** | ***P*** |
| Q̇_VL_ vs V̇O_2_ |  |  |  |  |  |  |
| Constant | 0.009 | 0.006 | -0.006 to 0.024 |  | 1.477 | 0.190 |
| V̇O_2_ | 0.085 | 0.011 | 0.058 to 0.112 | 1.859 | 7.743 | < 0.001* |
| V̇O_2_^^2^ | -0.015 | 0.004 | -0.026 to -0.005 | -0.804 | -3.590 | 0.012* |
| FM | -0.011 | 0.010 | -0.035 to 0.014 | -0.245 | -1.042 | 0.337 |
| FM*V̇O_2_ | 0.030 | 0.021 | -0.021 to 0.082 | 0.836 | 1.431 | 0.202 |
| FM* V̇O_2_^^2^ | -0.022 | 0.010 | -0.046 to 0.002 | -0.885 | -2.247 | 0.066 |
| Q̇_VL_ vs Q̇ |  |  |  |  |  |  |
| Constant | -0.030 | 0.026 | -0.094 to 0.033 |  | -1.177 | 0.284 |
| Q̇ | 0.015 | 0.005 | 0.002 to 0.027 | 2.026 | 2.817 | 0.030* |
| Q̇^2^ | -3.244e^-4^ | 2.414e^-4^ | -9.151e^-4^ to 2.663 e^-4^ | -0.957 | -1.344 | 0.228 |
| FM | -0.043 | 0.048 | -0.160 to 0.074 | -0.996 | -0.899 | 0.403 |
| FM* Q̇ | 0.008 | 0.010 | -0.015 to 0.032 | 2.171 | 0.853 | 0.427 |
| FM* Q̇^2^ | -4.903e^-4^ | 4.556e^-4^ | -16.95e^-4^ to 6.246e^-4^ | -1.676 | -1.076 | 0.323 |
| Q̇_VL_ vs MAP^a^ |  |  |  |  |  |  |
| Constant | -0.588 | 0.334 | -1.377 to 0.202 |  | -1.761 | 0.122 |
| MAP | 0.009 | 0.006 | -0.004 to 0.023 | 4.814 | 1.643 | 0.144 |
| MAP^2^ | -3.175e^-5^ | 2.480e^-5^ | -9.039e^-5^ to 2.689e^-5^ | -3.771 | -1.280 | 0.241 |
| FM | 0.035 | 0.044 | -0.070 to 0.140 | 0.813 | 0.790 | 0.455 |
| FM*MAP | -4.393e^-4^ | 3.759 e^-4^ | -13.283e^-4^ to 4.497 e^-4^ | -1.217 | -1.169 | 0.281 |
| Q̇_VL_ vs SVR |  |  |  |  |  |  |
| Constant | 0.206 | 0.034 | 0.123 to 0.289 |  | 6.070 | 0.001 |
| SVR | -0.014 | 0.006 | -0.030 to 0.001 | -1.716 | -2.247 | 0.066 |
| SVR^2^ | 2.416e^-4^ | 2.795e^-4^ | -4.423e^-4^ to 9.255e^-4^ | 0.659 | 0.864 | 0.421 |
| FM | -0.118 | 0.059 | -0.262 to 0.027 | -2.729 | -1.995 | 0.093 |
| FM*SVR | 0.018 | 0.011 | -0.009 to 0.045 | 4.588 | 1.626 | 0.155 |
| FM*SVR^2^ | -7.18e^-4^ | 4.883e^-4^ | -19.13e^-4^ to 4.77e^-4^ | -2.321 | -1.470 | 0.192 |
| Q̇_VL_, vastus lateralis blood flow; V̇O_2_, oxygen uptake; FM, dummy variable (patients = 1, controls = 0); Q̇, cardiac output; MAP, mean arterial pressure; SVR systemic vascular resistance. | | | | | | |
